# Testosterone as a Biomarker of Adverse Clinical Outcomes in SARS-CoV-2 Pneumonia

**DOI:** 10.3390/biomedicines10040820

**Published:** 2022-03-31

**Authors:** Lorenzo Marinelli, Guglielmo Beccuti, Marco Zavattaro, Serena Cagnina, Iacopo Gesmundo, Chiara Bona, Chiara Lopez, Silvia Scabini, Francesca Canta, Simone Mornese Pinna, Tommaso Lupia, Cataldo Di Bisceglie, Federico Ponzetto, Fabio Settanni, Francesco Giuseppe De Rosa, Ezio Ghigo, Giovanna Motta

**Affiliations:** 1 Division of Endocrinology, Diabetes, and Metabolism, Department of Medical Sciences, University of Turin, 10126 Turin, Italy; guglielmo.beccuti@unito.it (G.B.); serena.cagnina@unito.it (S.C.); iacopo.gesmundo@unito.it (I.G.); chiara.bona@unito.it (C.B.); chiara.lopez@unito.it (C.L.); cataldo.dibisceglie@unito.it (C.D.B.); federico.ponzetto@unito.it (F.P.); ezio.ghigo@unito.it (E.G.); 2 Division of Endocrinology, University Hospital “Maggiore della Carità”, 28100 Novara, Italy; marco.zavattaro@med.uniupo.it; 3 Division of Infectious Diseases, Department of Medical Sciences, University of Turin, 10126 Turin, Italy; silvia.scabini@unito.it (S.S.); fcanta@cittadellasalute.to.it (F.C.); smornesepinna@cittadellasalute.to.it (S.M.P.); francescogiuseppe.derosa@unito.it (F.G.D.R.); 4 Unit of Infectious Diseases, Cardinal Massaia Hospital, 14100 Asti, Italy; tommaso.lupia89@gmail.com; 5 Clinical Biochemistry Laboratory, AOU Città Della Salute e Della Scienza di Torino, 10126 Turin, Italy; fabio.settanni@unito.it

**Keywords:** SARS-CoV-2, COVID-19, pneumonia, testosterone, men, hypogonadism, hospitalization, mortality, fertility

## Abstract

Background: The severe acute respiratory syndrome coronavirus 2 (SARS-CoV-2) may affect testicles. Lower testosterone levels have been associated with worse clinical outcomes and higher mortality. Our objective was to evaluate the hypothalamic–pituitary–gonadal axis of men admitted with SARS-CoV-2 pneumonia and its link with the pneumonia-treatment intensification. Short-term changes in hormonal parameters were also assessed. Methods: Men admitted with SARS-CoV-2 pneumonia were recruited in two different hospitals in Piedmont, Italy. In all patients, the assessment of total testosterone (TT), calculated free testosterone (cFT), gonadotropins, inhibin B (InhB), and other biochemical evaluations were performed at admission (T0) and before discharge (T1). Through a review of medical records, clinical history was recorded, including data on pneumonia severity. Results: Thirty-five men (median age 64 [58–74] years) were recruited. Lower TT and cFT levels at T0 were associated with CPAP therapy (*p* = 0.045 and 0.028, respectively), even after adjusting for age and PaO_2_/FIO_2_ ratio in a multivariable analysis. In those discharged alive, lower TT and cFT levels were associated with longer hospital stay (*p* < 0.01). TT, cFT, and InhB were below the normal range at T0 and significantly increased at T1 (TT 1.98 [1.30–2.72] vs. 2.53 [1.28–3.37] ng/mL, *p* = 0.038; cFT (0.0441 [0.0256–0.0742] vs. 0.0702 [0.0314–0.0778] ng/mL, *p* = 0.046; InhB 60.75 [25.35–88.02] vs. 77.05 [51.15–134.50], *p* < 0.01). Conclusions: Both TT and cFT levels are associated with adverse clinical outcomes in men admitted with SARS-CoV-2 pneumonia. As TT, cFT and InhB levels increase before discharge, short-term functional recovery of steroidogenesis and an indirect improvement of spermatozoa functional status could be hypothesized.

## 1. Introduction

At the end of 2021, the severe acute respiratory syndrome coronavirus 2 (SARS-CoV-2) infected 27 million people globally, with more than 5.6 million deaths [1]. Its clinical manifestations are heterogeneous: beyond the well-known effects of the virus on the respiratory tract, smaller percentages of patients also reported gastrointestinal [2], neurological [3] and cardiovascular symptoms [4]. The host cell entry mechanism of SARS-CoV-2 has been thoroughly studied. The virus presents a surface-anchored spike protein containing a receptor-binding domain (RBD) that specifically recognizes angiotensin-converting enzyme 2 (ACE2) as its receptor [5]. Moreover, to fuse viral and lysosomal membranes, SARS-CoV-2 spike protein needs to be proteolytically activated by transmembrane protease serine 2 (TMPRSS2) and lysosomal proteases cathepsins [6,7]. Due to the widespread co-expression of ACE 2 and TMPRSS2 in several tissues, SARS-CoV-2 gain access to many different body areas such as the lungs, nose, brain, intestine, heart, kidneys, fallopian tubes, and testes [8,9,10]. In particular, it has been hypothesized that in the testis, local inflammatory response to SARS-CoV-2 could impair Leydig cell function, blood–testis barrier and directly damage seminiferous epithelium [11]. Therefore, potential consequences on spermatogenetic and steroidogenic functions may occur [12]. Beyond these direct mechanisms, levels of testosterone can be negatively influenced by indirect factors linked to SARS-CoV-2 infection as corticosteroid therapies or disease-related health impairment, such as obesity, hypertension, diabetes mellitus, which are known causes of functional hypogonadism [13,14,15,16]. Furthermore, these metabolic comorbidities in SARS-CoV-2 pneumonia were linked to a worse prognosis [17,18].

Testosterone levels seem to be involved in disease progression and severity [19,20]; in fact, testosterone decreases pro-inflammatory cytokines (IL-1 beta, IL-6, TNF-alpha), exerting an anti-inflammatory effect [21,22]. It is known that testosterone levels regularly decrease in men in their mid-30s and continue at an average rate of 1.6% per year [23]. Males aged over 65 years had major risks of complications from SARS-CoV-2 infection [24]. Some authors found a link between testosterone levels and clinical outcomes in admitted patients with COVID-19 pneumonia [25,26]. In particular, Rastrelli et al. found that lower baseline testosterone levels seemed to predict poor prognosis and mortality outcome in SARS-CoV-2-pneumonia men admitted to respiratory care unit [22]; another Italian group reported in COVID-19 infected men lower testosterone levels in patients with severe pneumonia in comparison with mild disease [27].

Even though a follow-up study involving SARS-CoV-2 pneumonia patients highlighted that testosterone levels increased over 7 months after recovery, little is known about impairment of the hypothalamic–pituitary–gonadal axis in men with this disease [28].

The aim of this prospective, multicentric study was to evaluate the hypothalamic–pituitary–gonadal axis of men admitted with SARS-CoV-2 pneumonia and its link with the pneumonia-treatment intensification. Additionally, short-term changes in hormonal parameters were assessed during hospitalization.

## 2. Materials and Methods

### 2.1. Study Population

A prospective, multicentric study was performed at “Città della Salute e della Scienza” University Hospital in Turin, Italy and “Cardinal Massaia” Hospital in Asti, Italy. Male patients were consecutively enrolled in general wards from March through June 2021. To be included in the study, patients had to be affected by SARS-CoV-2 pneumonia, defined by a positive nasopharyngeal swab and a chest X-ray or computer tomography consistent with interstitial pneumonia.

Clinical history was collected for each subject, focusing on cardiovascular comorbidities (arterial hypertension, diabetes mellitus, obesity), COPD, and the presence of other relevant diseases according to Charlson Comorbidity Index (CCI) [29]. The number of days from symptoms onset to hospital admission and a ratio of arterial oxygen partial pressure to fractional inspired oxygen concentration (PaO_2_/FiO_2_) were also recorded. Acute respiratory distress syndrome (ARDS) was defined by the Berlin criteria using PaO2/FiO2; severe ARDS was characterized as PaO_2_/FiO_2_ < 100 mmHg, moderate ARDS as Pao2/FiO2 100–200 mmHg, and mild ARDS as Pao2/FiO2 200–300 mmHg [30].

The first morning after hospitalization (T0) and the last day before discharge (T1), blood samples were drawn before 8 AM after overnight fasting to assess:

- a hormonal profile including total testosterone (TT), sex hormone-binding globulin (SHBG), luteinizing hormone (LH), follicle-stimulating hormone (FSH), 17-β estradiol (E2), albumin (ALB), inhibin B (InhB), Prolactin (PRL), 25OH vitamin D (25OHD), and prostatic serum antigen (PSA);
- an inflammatory/biochemical profile including blood count with lymphocytes cells count, C-reactive protein (CRP), procalcitonin (PCT), lactate dehydrogenase (LDH), ferritin, D-dimer, and fibrinogen.

Moreover, calculated free testosterone (cFT) was determined by Vermeulen formula using TT, SHBG and ALB levels [31].

Data about the clinical course of SARS-CoV-2 pneumonia, comprehensive of pharmacological support therapy (steroids—considered as dexamethasone or equivalent—heparin, antiretroviral—remdesivir—and immunomodulant therapy—tocilizumab), and oxygen support therapy (nasal cannula or Ventimask, CPAP, HFNC), were collected.

Written informed consent was obtained from all patients. This study was approved by the Local Ethical Committee (Studio CORACLE PROT.N. 0036628 16/02/2021).

### 2.2. Statistical Analysis

Continuous skewed variables are presented as median [25th–75th percentiles]. The Wilcoxon matched-pairs signed-rank test was conducted to highlight any difference in variables between T0 and T1. The Mann–Whitney U test was used for skewed data, while Fisher’s exact test was used for categorical variables. Spearman coefficients of correlation were also performed. Multivariable analyses were conducted using both a linear and logistic regression mode. The statistical analysis was conducted using the IBM SPSS program (IBM SPSS Statistics for Windows, Version 24.0. Armonk, NY, USA: IBM Corp). The statistical significance level was set at *p* < 0.05.

## 3. Results

### 3.1. Baseline Assessment/Admission Evaluation

Thirty-five patients were included in the study; the median age was 64 [58–74] years. The median time interval between the onset of respiratory symptoms and hospital admission was 8 [6–11] days.

The population characteristics are summarized in Table 1, including comorbidities; 40% of individuals had a CCI score >4, resulting in an estimated 10-year survival of 53%.

At admission, 94% of patients presented with mild ARDS, 6% with moderate ARDS, and no one with severe ARDS; the median PaO_2_/FiO_2_ was 271 [238–305].

An inflammatory pattern was observed, in line with active viral infection (CRP: 58.2 [22.9–136.7] mg/L, LDH: 659 [500–852.25] mg/dL, ferritin 1098.5 [634–1983.25] mg/dL, D-dimer 1030 [429–1703] mg/dL, and LYM 0.94 [0.57–1.14] cells/mm^3^).

Median TT (1.98 [1.06–2.67] ng/mL) and cFT levels (0.0415 [0.0239–0.0704]) were below the normal range, in accordance with a hypogonadal status [32]. Moreover, InhB (62.85 [33.02–91.4]) and 25OHD (11.2 [7.3–21.4]) were below the reference limits.

The subgroup with a higher CCI score presented had lower TT and cFT levels. Higher TT levels were correlated with lower PCT levels (rho = −0.375, *p* = 0.0314).

### 3.2. Hospital Stay Analysis

During hospitalization, 94% of individuals were treated with steroids and 89% with heparin. The prescription criteria for remdesivir (SARS-CoV-2 pneumonia within 10 days of the onset of symptoms, not requiring HFNC) and tocilizumab (COVID-19 pneumonia rapidly worsening after a starting dexamethasone treatment, with high levels of CRP) were fulfilled in 46% and 21%, respectively. Initial supplemental oxygen therapy via nasal cannula/Ventimask was used in 97% of patients; subsequently, 66% and 57% required CPAP therapy and HFNC, respectively.

CPAP use, considered an adverse clinical outcome, was associated with lower levels of TT (*p* = 0.045) and cFT (*p* < 0.03) at T0. After adjusting for age and PaO_2_/FIO_2_ at admission, both TT and cFT levels were inversely associated with CPAP use (multivariable logistic regression analysis, Table 2 and Table 3).

**Figure 1:**
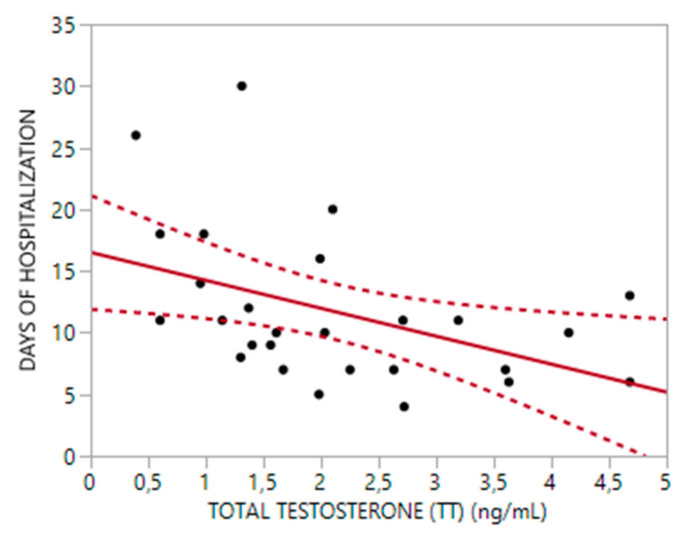
Relationship between Total Testosterone (TT) and Hospitalization time (R^2^ = 0.19, *p*: 0.021).

**Figure 2:**
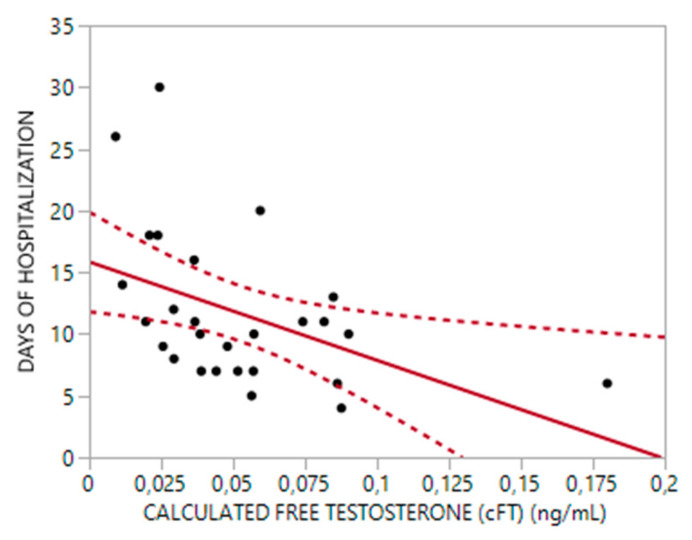
Relationship between calculated Free Testosterone (cFT) and Hospitalization time (R^2^ = 0.2, *p*: 0.018).

In the alive subgroup, longer hospitalization stays were significantly correlated with lower levels of TT and cFT (rho = −0.51, *p* < 0.01 and rho = −0.55, *p* < 0.01, respectively) (Figure 1 and Figure 2), as well as older age (rho = 0.5, *p* < 0.01) and higher CCI score (rho = 0.60, *p* < 0.01). A multiple regression analysis showed that TT levels were independent predictive factors for days of hospitalization; about 32% of the variance in admission days (Adjusted R^2^: 32%, *p* < 0.01) could be accounted for TT (Beta = −1.99, 95%CI = −3.72; −0.27 *p* < 0.03) and age (Beta = 0.23, 95%CI = 0.05–0.41, *p* < 0.02).

Table 4 summarizes the characteristics of alive patients at T0 and T1. Median time between T0 and T1 was 10 [7–13] days. At baseline, median TT (1.98 [1.30–2.72] ng/mL) and cFT (0.0475 [0.0253–0.0824] ng/mL) were below the normal range values. During hospital stay, median TT and cFT levels increased (TT 1.98 [1.30–2.72] vs. 2.53 [1.28–3.37] ng/mL; *p* = 0.038 and cFT (0.0475 [0.0253–0.0824] vs. 0.0702 [0.0314–0.0778] ng/mL; *p* = 0.046). LH levels decreased (5.3 [3.20–7.10] vs. 2.9 [2.10–5.6]; *p* < 0.01), while no difference in FSH levels were observed. Moreover, InhB levels showed an increase (60.75 [25.35–88.02] vs. 77.05 [51.15–134.50]; *p* < 0.01). SHBG (25.8 [18.4–36.1] vs. 24.65 [16.75–33.05]; *p* = 0.046), and PRL (12 [8.1–16] vs. 16.9 [9.3–23.5]; *p* < 0.01) raised during observation time.

E2, 25OHD and PSA did not show any differences between T0 and T1.

### 3.3. Mortality Evaluation

In-hospital mortality rate was 20%. Deceased patients showed a higher CCI and higher levels of CRP and PCT (0.42 [0.14–0.63] vs. 0.10 [0.06–0.18]; *p* = 0.0175). No hormonal differences were observed between alive and dead individuals; specifically, in deceased patients TT and cFT were lower (TT 1.55 [0.77–2.29] vs. 1.98 [1.30–2.72]; cFT 0.0222 [0.0134–0.0703] vs. 0.0441 [0.0256–0.0742]) but the difference did not reach statistical significance.

None of the hormonal parameters was associated with mortality in a logistic regression model (data not shown).

## 4. Discussion

The present study shows that, in men admitted with SARS-CoV-2 pneumonia, TT and cFT levels were below the normal value range at admission, with a higher probability of hypogonadal symptoms, in accordance with current Endocrine Society guidelines on male hypogonadism [32]. Moreover, TT and cFT levels appeared to be related to adverse clinical outcomes such as longer hospitalization days and the necessity of pneumonia-treatment intensification (CPAP therapy).

SARS-CoV-2 infection can be characterized by a systemic involvement; in fact, aside from the respiratory tract, the endocrine system is susceptible to SARS-CoV-2, particularly the hypothalamic–pituitary–gonadal axis. To date, it is unclear whether the observed impairment of the gonadal function is due to a primary testicular injury [33] or to a hypothalamic–pituitary dysfunction [34].

It has been demonstrated that Sertoli and Leydig cells express ACE2 receptor [35,36] primarily used by SARS-CoV-2 for penetration into cells, while TMPRSS2, which is also involved in the viral entry, is found in primordial spermatozoa [37]. Thereby, direct colonization of the virus in the testis can be speculated, with a potential gonadal impairment induced by the release of pro-inflammatory chemokines and cytokines, also known as “cytokine storm” [38]. In our study, LH levels were in the upper limit of the normal range; this would resemble a hormonal status similar to primitive hypogonadism, suggesting an impairment in testicular function. An orchitis-like syndrome has been hypothesized in SARS-CoV-2 infection [39] as reported in other SARS-CoV and different viruses, such as Zika, mumps, and human papilloma virus [40]. Some authors succeeded to document ultrasound findings of acute orchitis, epididymitis and epididymo-orchitis in men infected by SARS-CoV-2 [41]. Unfortunately, our findings were not implemented by a testicular ultrasound evaluation, which could have provided more speculative data.

Conversely, an impairment in hypothalamic–pituitary function might not be excluded. One of the cornerstones of SARS-CoV-2 pneumonia therapy is represented by high dosage steroids (e.g., dexamethasone 6 mg daily) [42], which is known to cause gonadotropin hormone-releasing hormone (GnRH) dysregulation and subsequently secondary hypogonadism [43]. Previous literature has shown how systemic disorders not related to COVID-19 (e.g., cancer, rheumatic, and end-stage diseases) imply a chronic inflammatory status. This is responsible for the hypothalamic–pituitary–gonadal axis impairment, known as functional hypogonadism [32,44,45,46]. In fact, in the present study, individuals with a higher number of comorbidities (represented by a higher CCI) showed lower TT and cFT levels.

Moreover, COVID-19 pneumonia can be characterized by an extensive lung impairment, leading to significant hypoxemia. It has to be considered that in men affected by chronic hypoxemia, such as in chronic obstructive pulmonary disease (COPD) [47] or in obstructive sleep apnea syndrome (OSAS) [48], hypogonadism can be observed. Lower testosterone levels and higher levels of LH have been reported in men with COPD [47] and in particular during COPD exacerbation [49]. Patients with OSAS show a hypoxia-driven decrease in LH and testosterone levels [48], associated with an alteration of the circadian rhythm of testosterone secretion [50], with normalization after CPAP treatment [51].

In summary, the highlighted hypogonadal status could be explained by a contribution of both primary and secondary mechanisms.

TT and cFT levels, but not other hormonal parameters, were inversely associated with inpatient days. Although the hospitalization length was positively correlated to age and consequently to CCI, our multivariable model showed that the inverse association between gonadal steroids and prolonged hospital stay was independent of age. It is noteworthy that this analysis included only those discharged alive, with limited statistical power. Our findings are consistent with other studies that highlighted this inverse link between testosterone and hospitalization days [27,52].

In the whole group, even though most of the patients presented with a mild ARDS (median PaO_2_/FIO_2_ ratio = 271), 66% of them eventually required CPAP therapy. Both TT and cFT values were significantly associated with CPAP therapy, even after adjusting for PaO_2_/FIO_2_ ratio and age, as testosterone levels physiologically decrease by 2% yearly by the age of 35 due to tissue senescence [23]. These findings suggest that testosterone levels could be considered an independent marker of severity and worsening of respiratory outcomes. Other oxygen support therapies, such as nasal cannula and HFNC, did not show the same positive association. The relationship between testosterone and clinical outcomes has also been described by other authors, who highlighted how lower testosterone levels are associated with a more severe clinical illness that requires intensive care [22,25,53].

COVID-19 infection shows a significant mortality rate [54], especially in older men suffering from a higher number of comorbidities [24]. In our cohort, 20% of individuals died of SARS-CoV-2 pneumonia. Even though TT and cFT were lower in deceased patients, a statistically significant difference was not highlighted when compared with the alive. At variance with other studies [25,34,55], TT and cFT levels, as well as other hormonal parameters, did not predict mortality. This could be probably due to the small sample size of dead individuals and to other confounding factors.

To date, this is the first study assessing the short-term changes in hypothalamic–pituitary–gonadal axis function in hospitalized men with SARS-CoV-2 pneumonia. After a median hospital stay of 10 days, TT and cFT levels improved and reached the low-normal value range, according to current Endocrine Society guidelines [32]. Moreover, serum LH significantly decreased, as could be expected after recovering from a testicular injury. A recent study on men with a SARS-CoV-2 positive nasopharyngeal swab but unspecified radiological lung involvement showed low TT values at enrollment [53]. An increase in TT levels was observed after 7 days from diagnosis in mild disease and after a month in severe disease; no difference in estradiol levels was reported. Unfortunately, LH measurement was not performed.

Interestingly, in our sample, InhB levels were low at admission and raised after 10 days of hospitalization, while FSH did not show a significant variation. InhB is a gonadal glycoprotein predominantly secreted by Sertoli cells [56,57], and it is considered to be a marker of the functional state of the seminiferous epithelium. Moreover, InhB seems to be positively associated with spermatogenesis, better than FSH [58,59,60], with a particularly strong correlation with low sperm counts [61]. The aforementioned hypothesis of the direct viral colonization is reasonably supported by the expression of ACE2 receptor in Sertoli cells and TMPRSS2 in spermatogonial stem cells, elongated spermatids, and to a lesser extent in stem cells [37]. Previous studies, however, did not report any viral RNA in semen samples, except for one study in alive patients [2] and one in post-mortem examination of testicular specimens [62]. Nevertheless, sperm samples collected after recovering from COVID-19 infection showed a wide spectrum of alterations involving semen volume, sperm concentration, morphology, motility, and DNA fragmentation [33,63]. Even though semen samples were not collected, data from the present study indirectly seems to suggest a conceivable spermatogenic impairment at admission for SARS-CoV-2 pneumonia and give a reassuring clue about the short-term improvement of spermatogenic cells function. In conclusion, this short-term reversibility supports the hypothesis that this gonadal impairment could be mainly related to COVID-19 infection rather than the burden of comorbidities.

This study presents some limitations. First, it is not possible to draw definitive conclusions about causality due to the observational design of the study and the several comorbidities that could interfere with the hypothalamic–pituitary–gonadal axis. Second, the sample size is relatively small, particularly after stratification by mortality. Third, hormonal evaluations were not available before SARS-CoV-2 infection and pneumonia development, as well as long-term ones after discharge. Lastly, the lack of a control group with different types of pneumonia or infections could represent an issue but opens a new, intriguing scenario where it is conceivable to test TT and cFT as biomarkers of adverse clinical outcomes, also in non-SARS-CoV-2 settings.

Before suggesting a routine assessment of the gonadal function in individuals admitted with SARS-CoV-2 pneumonia, future studies are warranted to extensively evaluate the role of androgen levels as an early predictive test for lung disease severity.

## 5. Conclusions

This multicenter observational study on men admitted with SARS-CoV-2 pneumonia shows that both TT and cFT levels appeared to be related to adverse clinical outcomes such as longer hospitalization and the need for pneumonia-treatment intensification, independent of age. Furthermore, TT, cFT and InhB levels rise before discharge, suggesting a short-term functional recovery of steroidogenesis and an indirect improvement of spermatozoa functional status. In accordance with previous studies, gonadal status could be considered as a new, simple biomarker to early identify patients with a higher probability of worse clinical outcomes, but it has still to be validated, both in SARS-CoV-2 and non-SARS-CoV-2 settings.

## Figures and Tables

**Table 1:** Demographic data, comorbidities, and ARDS severity at admission of the whole cohort.

Age (Years)	64 [58–74]
BMI (Kg/m^2^)	29.65 [25.55–30.87]
**CCI (score)**	
0–1 (%)	28.6
2–3 (%)	31.4
≥4 (%)	40
**Smoking habits**	
Current smoker (%)	8.6
Former smoker (%)	60.8
**Main comorbidities**	
COPD (%)	21
Arterial hypertension (%)	40
Diabetes (%)	15
Obesity (%)	21
**ARDS severity at admission**	
PaO_2_/FiO_2_ ratio	271 [238–305]
mild ARDS ^a^ (%)	94
moderate ARDS ^b^ (%)	6
severe ARDS ^c^ (%)	0

Data are presented as median (interquartile range). BMI = Body Mass Index, CCI: Charlson Comorbidity Index, COPD: Chronic Obstructive Pulmonary Disease, ARDS: Acute Respiratory Distress Syndrome. ^a^ (PaO_2_/FiO_2_ 200–300 mmHg), ^b^ (PaO_2_/FiO_2_ 100–200 mmHg), ^c^ (PaO_2_/FiO_2_ <100 mmHg).

**Table 2:** Multivariable logistic regression analysis for CPAP Therapy using TT.

	OR	95% CI	*p* *
**AGE**	0.999	0.883; 1.131	0.990
**TT**	0.109	0.0129; 0.916	<0.001
**PaO_2_/FiO_2_**	0.950	0.915; 0.987	<0.001

* Statistical significance levels at *p* < 0,05. OR: Odds Ratio, CI: Confidence Interval, TT: Total Testosterone, PaO_2_/FiO_2_: ratio of arterial oxygen partial pressure to fractional inspired oxygen concentration.

**Table 3:** Multivariable logistic regression for CPAP Therapy using cFT.

	OR	95% CI	*p* *
**AGE**	0.974	0.873; 1.086	0.624
**cFT**	0.450	0.209; 0.969	0.001
**PaO_2_/FiO_2_**	0.953	0.917; 0.989	<0.001

* Statistical significance levels at *p* < 0,05. OR: Odds Ratio, CI: Confidence Interval, cFT: calculated Free Testosterone, PaO_2_/FiO_2_: ratio of arterial oxygen partial pressure to fractional inspired oxygen concentration.

**Table 4:** Biochemical and hormonal assessment between admission (T0) and discharge (T1).

Biochemical Assessment	Admission (T0)	Discharge (T1)	*p*-Value *
WBC (10^9^/L)	7.58 [4.92–12.98]	9.12 [6.417–12.452]	0.572
LYM (10^9^/L)	0.94 [0.57–1.14]	1.15 [1.355–2.26]	<0.001
PLT (10^9^/L)	227 [177–307]	243 [198.7–352]	0.102
CRP (mg/L)	58.2 [22.9–136.7]	8 [3.3–12.3]	<0.001
PCT (ng/mL)	0.14 [0.06–0.42]	0.09 [0.045–0.46]	0.028
D-DIMER (ng/mL)	1030 [429–1703]	1050 [298–1560]	0.219
LDH (IU/L)	659 [500–852.25]	458 [391–710.5]	0.001
FERRITIN (mg/dL)	1098 [634–1983.25]	796 [453.5–1252.5]	<0.001
**Hormonal parameters**			
TT (ng/mL)	1.98 [1.30–2.72]	2.695 [1.26–3.43]	0.038
cFT (ng/mL)	0.0441 [0.0256–0.0742]	0.0702 [0.0314–0.0778]	0.017
E2 (pg/mL)	22 [19–34.20]	18.75 [14.75–30.25]	0.131
LH (UI/L)	5.3 [3.20–7.10]	2.83 [2.02–5.5]	<0.001
FSH (UI/L)	4.9 [3.40–7.40]	4.45 [3.30–8.97]	0.591
InhB (pg/mL)	60.75 [25.35–88.02]	77.05 [51.15–134.50]	<0.001
SHBG (nmol/L)	25.8 [18.4–36.1]	24.65 [16.75–33.05]	0.099
ALB (g/dL)	3.4 [3.07–3.62]	3.45 [3.02–3.70]	0.202
PRL (ng/mL)	12 [8.1–16]	17.05 [9.8–23.67]	0.002
25OHD (ng/mL)	12.5 [7.7–23.8]	16 [12.7–22.17]	0.667
PSA (ng/mL)	0.8 [0.4–2.7]	1.25 [0.57–2.37]	0.473

* Statistical significance levels at *p* < 0.05. Data are presented as median (interquartile range). WBC: White Blood Cell, LYM: Lymphocytes, PLT: Platelets CRP: C-Reactive Protein, PCT: Procalcitonin, LDH: Lactate Dehydrogenase, TT: Total Testosterone, cFT: calculated Free Testosterone, E2: 17-β estradiol, LH: Luteinizing Hormone, FSH: Follicle Stimulating Hormone, InhB: Inhibin B, SHBG: Sex Hormone Binding Globulin, ALB: albumin, PRL: Prolactin, 25OHD: 25 OH vitamin D, PSA: Prostatic Serum Antigen (PSA).

## Data Availability

The data presented in this study are available on request from the corresponding author. The data are not publicly available due to the local ethics committee policy.
